# Phenylsilane as an effective desulfinylation reagent

**DOI:** 10.3762/bjoc.13.150

**Published:** 2017-08-01

**Authors:** Wanda H Midura, Aneta Rzewnicka, Jerzy A Krysiak

**Affiliations:** 1 Centre of Molecular and Macromolecular Studies, Polish Academy of Sciences, Department of Heteroorganic Chemistry, Sienkiewicza 112, 90-363 Łódź, Poland

**Keywords:** desulfinylation, phenylsilane, reduction, regioselectivity, α-sulfinylesters

## Abstract

The reduction using phenylsilane in a KOH-catalyzed system was applied successfully to the conversion of sulfinyl-substituted cyclopropylcarboxylates into the corresponding alcohols. The presence of sulfinyl substituents in the α-position to the carboxylate group caused a desulfinylation product formation with full regio- and stereoselectivity, instead of a carbonyl group reduction. Investigations performed on different α-sulfinylcarbonyl compounds revealed that phenylsilane treatment constitutes a regiospecific method for the desulfinylation of a-sulfinylesters; for corresponding ketones the reaction course depends on the character of the carbonyl group.

## Introduction

The conversion of carboxylic acid derivatives to the corresponding alcohols is one of the fundamental processes in organic synthesis. Over the past decades, many reagents and conditions for this transformation have been reported. Apart from traditional hydride reducing agents like lithium aluminum hydride and sodium borohydride [1–2], different modifications were applied, using transition metal salts as catalysts or additives to change or enhance the properties of these reagents [3–8]. Hydrogenation is another convenient method for the reduction of carbonyl compounds, although low selectivities have sometimes been observed and the reaction usually required drastic conditions [9]. Also, the catalytic hydrosilylation of carbonyl moieties has become an important transformation as an alternative reduction methodology [10–14]. Besides the well-known metal-catalyzed hydrosilylation, transition metals in the presence of Brønsted or Lewis acids were used for the reduction. Recently base-activated silanes were also used for this purpose [15–17].

## Results and Discussion

**Scheme 1:**
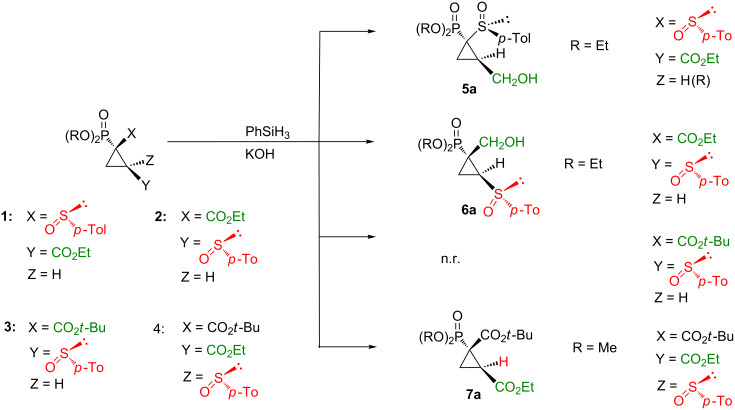
Different behaviour of cyclopropylphosphonates in the reaction with phenylsilane.

Our recent investigations were connected with the synthesis and further conversion of multifunctional cyclopropanes, where the carboxylate moiety was present in close proximity to sulfinyl and phosphoryl substituents [18–20]. A selective and effective method of the independent conversion of each of these substituents is therefore a subject of our interest. To extend the investigations on the reduction of esters using a reagent with tolerance for other functional groups present in the structure, we applied a hydrosilylation methodology (Scheme 1). Various cyclopropyl phosphonates were subjected to the reaction with an excess of phenylsilane under neat reaction conditions in the presence of a catalytic amount of KOH. It appeared that results of this reaction strongly depended on the relative position of particular substituents [21].

For compounds **1** and **2** the treatment with phenylsilane caused, as expected, in a reduction of the ester group. In both cyclopropanes the carboxylate moiety and the sulfinyl group were in vicinal relation, but in **1**, formed in the reaction of vinyl phosphoryl sulfoxide with EDSA [22], the ester group was easily accessed, since the bulky phosphoryl group was also in vicinal position to the sulfoxide substituent. Compounds **2** and **3** were obtained by us earlier [23] in diastereomerically pure form as key intermediates in our approach to constrained phosphonic acids by the reaction of phosphoryl acrylates with (*S*)-dimethylsulfonium(*p-*tolylsulfinyl)methylide. The carboxylate moiety was geminal to the phosphoryl substituent in these compounds. In the case of cyclopropane **3** the reduction did not occur, since the bulky *tert*-butyl substituent effectively protected the carboxylate group. Hence, the starting material was recovered.

The next cyclopropane **4**, which contained the sulfinyl substituent in geminal position to the ester group, was subjected to a reaction with phenylsilane using the procedure described above. In this case, to our surprise, no alcohol was detected, but the desulfinylation process occurred exclusively.

Cyclopropane **4** was formed by carboxylation of **3** with ethyl chloroformate as a continuation of our studies on asymmetric cyclopropanation of vinyl phosphonates using (*S*)-dimethylsulfonium(*p-*tolylsulfinyl)methylide and its further application. It was formed as a single diastereomer with full stereoselectivity. To determine the relative configuration of the obtained structure, in the absence of an α-proton at the sulfinyl group which was conclusive in our former studies, we benefited from ^13^C NMR analysis. The small value of coupling constant ^3^*J*_P-C_ = 4 Hz indicated a *trans* relationship between the carboxylate and the phosphoryl substituent.

**Scheme 2:**
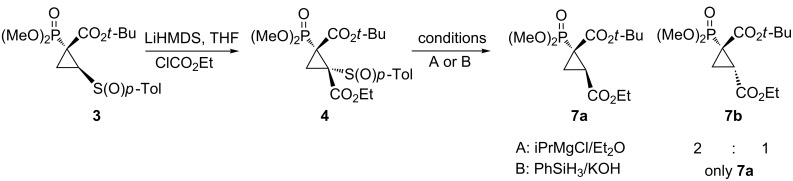
Synthesis and desulfinylation of **4**.

Desulfinylation of cyclopropane **4** under conditions used by us before, i.e., by treatment with isopropylmagnesium chloride, gave a mixture of diastereomers **7a** and **7b**. It was probably due to epimerization on carbon C2 of the carbanion, formed after attack of the Grignard reagent on sulfur. To our satisfaction, utilization of the silane procedure led to the single diastereomer **7a**. In this case determination of the relative configuration as *trans* was based on a high coupling constant ^3^*J*_P-H_ = 17.0 Hz, which evidenced retention of configuration during the desulfinylation process (Scheme 2).

The reaction conducted under solvent-free conditions with a large excess of silyl reagent (procedure 1) occurred with full conversion of the cyclopropanes, although some problems with the isolation of the product due to an excess of silane induced a modification of the reduction procedure. The gain of using THF as a solvent [17] (procedure 2) let us decrease the amount of phenylsilane used in the reaction medium, which resulted in a high yield of the corresponding cyclopropane **7a**.

**Scheme 3:**
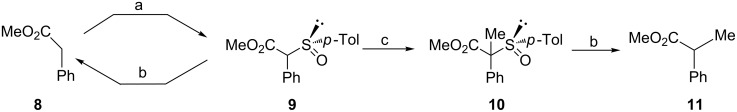
Reaction of acyclic sulfoxides with phenylsilane. Reagents and conditions: (a) BuLi, THF, −70 °C, *p*-TolS(O)Men; (b) PhSiH_3_, THF, rt, KOH (5 mol %); (c) MeI, 50% KOH, CH_2_Cl_2_.

The obtained results demonstrated that the presence of a sulfinyl substituent in α position to the carboxylate completely changed the direction of the silane attack on the structure, so this reaction could be considered as a new regiospecific method of a desulfinylation. In order to examine the scope of observed desulfinylation different carbonyl compounds with sulfinyl substituents were synthetized and subjected to the reaction with phenylsilane. Reactions of α-sulfinylcarboxylates **9** and **10** with phenylsilane evidenced that the desulfinylation process occurred also in the case of acyclic sulfoxides (Scheme 3).

α-Sulfinylphenylacetate **9** was formed as a mixture of diastereomers difficult to separate. This mixture, without separation, was subjected to the reaction with phenylsilane utilizing the procedure described above and afforded phenylacetate **8** as the only product. Methylation of **9** afforded product **10** with full stereoselectivity as the only diastereomer although the configuration of this center was not determined. Also in this case treatment with PhSiH_3_/KOH caused a desulfinylation process leading to ester **11** [24]. Unfortunately in this case the stereogenic center at the carbon undergoes epimerization under these reaction conditions and **11** is obtained in racemic form.

To study the scope and limitation of the silane reactivity and its selectivity we checked the influence of the ketone function on the behaviour of the compounds with sulfur and carbonyl substituents in geminal position. This relation turned out to be more complex and was depended on the ketone structure (Table 1).

**Table 1:** Reaction of compounds with sulfur and carbonyl substituents in geminal relation with PhSiH_3_/KOH.

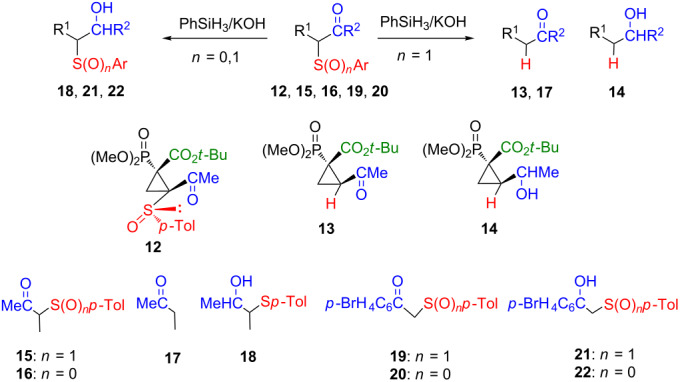				

Entry	Ketone	Reaction conditions	Product of reduction	Product of desulfinylation

1	**12**	5 equiv PhSiH_3_, 0.1 equiv KOH, 0.5 h, rt, 20% conv.	not detected	**13/14** 2:1
2	**12**	5 equiv PhSiH_3_, 0.1 equiv KOH, 5 h, rt, 100% conv.	not detected	**13/14** 2:1
3	**15**	5 equiv PhSiH_3_, 0.1 equiv KOH, 5 h, rt, 100% conv.	not detected	**17**
4	**16**	5 equiv PhSiH_3_, 0.1 equiv KOH, 5 h, rt, 100% conv.	**12**	not detected
5	**19**	5 equiv PhSiH_3_, 0.1 equiv KOH, 5 h, rt, 100% conv.	**21**	not detected
6	**20**	5 equiv PhSiH_3_, 0.1 equiv KOH, 5 h, rt, 100% conv.	**22**	not detected

Introduction of an acyl moiety by substitution of **3** with acetic anhydride led to compound **12**, which was subjected to the reaction with PhSiH_3_/KOH. Also in this case desulfinylation of cyclopropane took place leading to **13** as a major product, however, accompanied by the corresponding alcohol **14**, formed as a result of the subsequent reduction of the carbonyl group (Table 1, entries 1 and 2). Oxidation of the crude mixture by stirring with chromium trioxide and pyridine allowed to obtain desulfinylated ketone **13** in a reasonable yield.

For acyclic structures the reaction course dramatically depended on the character of ketone. In the case of aliphatic ketone **15**, silane treatment gave desulfinylated product **17** as the only product (Table 1, entry 3). However, the presence of a more electrophilic aryl ketone group in **19** caused that the reduction of carbonyl group took place, leading to alcohol **21** without desulfinylation (Table 1, entry 5).

For comparison, for analogous carbonyl structures with sulfide substituents, under these conditions, reduction of the carbonyl group occurred leading to the corresponding alcohols (**18** [25], **22**) as the only products in high yield. It means that attack on sulfur is not connected with the presence of a C–S bond but a sulfinyl substituent is required.

In a former research performed by Fernandes and Romão [26], an α-sulfinyl-substituted ester (methyl(phenylsulfinyl)acetate) was treated by phenylsilane, but using a silane/MoO_2_Cl_2_ system as a catalyst. Under those conditions both functional groups were reduced, leading to the corresponding α-phenylsulfenyl alcohol. The desulfinylation process of compounds of this type by phenylsilane is the first observation. It is unknown until now in literature. One could assume that the active species, usually postulated in the fluoride- and base-catalyzed hydrosilylation [27–30], in the presented case is additionally chelated by both polar carbonyl substituents. It facilitates hydride anion formation, which attacks the most electrophilic center of a particular structure.

## Conclusion

A phenylsilane/KOH system was successfully applied as a reducing agent for conversion of different cyclopropylcarboxylates to the corresponding alcohols. However, our study revealed that the presence of a sulfinyl substituent in the α-position to the carboxylate moiety totally changes the direction of the reaction, leading to the corresponding ester deprived of the sulfinyl group. The desulfinylation process of compounds of this type by phenylsilane was observed for the first time, so far unknown in the literature. This approach increases the number of selective desulfinylation methods which can be performed under mild conditions [31–33]. The reactivity of α-sulfinylketones with phenylsilane is less regioselective and the direction of the reaction strongly depends on the character of the ketone.

## Supporting Information

File 1 Experimental and analytical data and NMR spectra.

## References

[1] Seyden-Penne J (1997). Reductions by the Alumino- and Borohydrides in Organic Synthesis.

[2] Gribble G W (1998). Chem Soc Rev.

[3] Ganem B, Osby J O (1988). Chem Rev.

[4] Kanth J V B, Periasamy M (1991). J Org Chem.

[5] Narasimhan S, Madhavan S, Prasad K G (1995). J Org Chem.

[6] Reding M T, Buchwald S L (1995). J Org Chem.

[7] Jagdale A R, Paraskar A S, Sudalai A (2009). Synthesis.

[8] Morales-Serna J A, García-Rios E, Bernal J, Paleo E, Gaviño R, Cárdenas J (2011). Synthesis.

[9] Pouilloux Y, Autin F, Barrault J (2000). Catal Today.

[10] Marciniec B, Maciejewski H, Pietraszuk C, Pawluć P, Marciniec B (2009). Hydrosilylation. A Comprehensive Review on Recent Advances.

[11] Marciniec B (2005). Coord Chem Rev.

[12] Addis D, Das S, Junge K, Beller M (2011). Angew Chem, Int Ed.

[13] Marciniec B, Gulinsky J, Urbaniak W, Kornetka Z W, Marciniec B (1992). Comprehensive Handbook on Hydrosilylation.

[14] Ojima I, Patai S, Rappoport Z (1989). The Chemistry of Organic Silicon Compounds.

[15] Fedorov A, Toutov A A, Swisher N A, Grubbs R H (2013). Chem Sci.

[16] Fernández-Salas J A, Manzini S, Nolan S P (2013). Chem Commun.

[17] Revunova K, Nikonov G I (2014). Chem – Eur J.

[18] Midura W H, Cypryk M, Rzewnicka A, Krysiak J A, Sieroń L (2016). Eur J Org Chem.

[19] Midura W H, Krysiak J, Rzewnicka A, Supeł A, Łyżwa P, Ewas A M (2013). Tetrahedron.

[20] Krysiak J, Midura W H, Wieczorek W, Sieroń L, Mikołajczyk M (2010). Tetrahedron: Asymmetry.

[21] Midura W H, Rzewnicka A, Krysiak J A (2017). Phosphorus, Sulfur Silicon Relat Elem.

[22] Midura W H, Krysiak J, Mikołajczyk M (2003). Tetrahedron: Asymmetry.

[23] Midura W H, Rzewnicka A (2013). Tetrahedron: Asymmetry.

[24] Yamashita T, Yasueda H, Miyauchi Y, Nakamura N (1977). Bull Chem Soc Jpn.

[25] Sun J, Yang M, Yuan F, Jia X, Pan Y, Zhu C (2009). Adv Synth Catal.

[26] Fernandes A C, Romão C C (2006). J Mol Catal A: Chem.

[27] Lawrence N J, Drew M D, Bushell S M (1999). J Chem Soc, Perkin Trans 1.

[28] Kobayashi Y, Takahisa E, Nakano M, Watatani K (1997). Tetrahedron.

[29] Hojo M, Murakami C, Fujii A, Hosomi A (1999). Tetrahedron Lett.

[30] Hojo M, Fujii A, Murakami C, Aihara H, Hosomi A M (1995). Tetrahedron Lett.

[31] Capozzi M A M, Cardellicchio C, Naso F, Tortorella P (2000). J Org Chem.

[32] García Ruano J L, Marzo L, Marcos V, Alvarado C, Alemán J (2012). Chem – Eur J.

[33] Frutos M, Ortuño M A, Lledos A, Viso A, Fernández de la Pradilla R, de la Torre M C, Sierra M A, Gornitzka H, Hemmert C (2017). Org Lett.

