# Supplementary material for: Phenylsilane as an effective desulfinylation reagent
Source: Beilstein J Org Chem. 2017 Aug 1;13:1513–7. doi: 10.3762/bjoc.13.150 (PMC5550773; doi:10.3762/bjoc.13.150)

Supporting Information
for
Phenylsilane as an effective desulfinylation reagent

Wanda H. Midura,* Aneta Rzewnicka, Jerzy A. Krysiak

Address: Centre of Molecular and Macromolecular Studies, Polish Academy of Sciences, Department of Heteroorganic Chemistry, 90-363 Łódź, Sienkiewicza 112, Poland

Email: Wanda H. Midura - whmidura@cbmm.lodz.pl

*Corresponding author

Experimental and analytical data and NMR spectra

Table of contents

1. Experimental section S2
2. ^{1}H and $^{13}C\{1H\}$ NMR Spectra of the starting materials S8
3. ^{1}H and $^{13}C\{1H\}$ NMR Spectra of the Products S12

1. Experimental section

1.1. General

Glassware was dried prior to use by heating under vacuum. All air and water sensitive reactions were carried out under an argon atmosphere. Commercial grade reagents and solvents were used without further purification except as indicated below. THF and ethyl ether were freshly distilled over sodium/benzophenone Thin layer chromatography (TLC) was conducted on Silica Gel 60 F254 TLC. Column chromatography was performed using silica gel (70–230 mesh). ^{1}H, ^{13}C and ^{31}P NMR spectra were recorded on 500 and 200 spectrometer. The mass spectra and HRMS were measured using a double-focusing (BE geometry) mass spectrometer utilizing a chemical ionization (CI), with isobutane as an ionizating agent, or electron ionization (EI) technique as well as electrospray ionization technique. Optical rotations were measured using a photopolarimeter in acetone solution. Melting points were uncorrected. The microanalyses were performed on elemental analyzer.

1.2. General procedure for the acylation

To a solution of 0.2 mmol (67.9 mg) of cyclopropyl sulfoxide in THF (1.5 mL), LiHMDS (prepared by addition of 0.11 mL of 2 M solution of BuLi to 0.05 mL of HMDS) was added at −70 °C. The reaction mixture was stirred for 20 min and (0.3 mmol) of acylating reagent ($ClCO_2Et/Ac_2O$) was then added at −70 °C, and the reaction was allowed to warm slowly to 0 °C. After quenching with NH_4Cl the aqueous phase was extracted five times with CH_2Cl_2, and the combined organic solution was dried over $MgSO_4$, filtrated and evaporated.

1.2.1. (+)-(1*R*,2*S*,*S*s)-*tert*-Butyl 1-dimethylphosphono-2-*p*-tolylsulfinyl-2-carboethoxycyclo-propane carboxylate (4)

The crude product was purified by recrystallization from Et_2O. White solid; m.p. 115-117 ^{o}C; Yield 70 %; $[\alpha]_D^{20}$ +131.4 (1.2 acetone) ^{31}P NMR (81 MHz, $CDCl_3$) δ: 20.3 ppm; ^{1}H NMR (500 MHz, $CDCl_3$) δ: 1.03 (t, J_{HH} = 7.5 Hz, 3H, $CO_2CH_2C\underline{H}_3$), 1.41 (s, 9H, $COC(C\underline{H}_3)_3$), 2.12 (dd, J_{HH} = 5.8 Hz, J_{PH} = 15.6 Hz, 1H, $C\underline{H}_{cis}$), 2.28 (dd, J_{HH} = 5.8 Hz, J_{PH} = 9.8 Hz, 1H, $C\underline{H}_{trans}$), 2.39 (s, 3H, $C_6H_4C\underline{H}_3$), 3.82-3.85 (m, 1H, $POC\underline{H}_2CH_3$), 3.86 and 4.04 (2xd, J_{PH} = 11.3 Hz, 6H, $POC\underline{H}_3$,), 3.94–3.97 (m, 2H, $CO_2C\underline{H}_2CH_3$), 7.30 and 7.54 (A_2B_2, 4H, $C_6\underline{H}_4CH_3$) ppm; ^{13}C NMR (125 MHz, $CDCl_3$) δ: 19.4 (d, J_{CP} = 2.2 Hz, $\underline{C}H_2C$), 21.4 ($C_6H_4\underline{C}H_3$), 27.9 ($COC(\underline{C}H_3)_3$), 30.6 (d, J_{CP} = 180.6 Hz), 53.7 (d, J_{CP} = 5.8 Hz, $PO\underline{C}H_3$), 54.5 (d, J_{CP} = 5.8 Hz, $PO\underline{C}H_3$), 56.2 (d, J_{CP} = 3.3 Hz, $\underline{C}SO$), 83.7 ($\underline{C}OC(CH_3)_3$), 124.8, 129.6, 138.6, 142.0, 162.9

(d, J_{CP} = 4.3 Hz), 164.3 (d, J_{CP} = 4.3 Hz); MS(EI) 460; HRMS (EI) *m/z* calcd for $C_{20}H_{29}O_8PS$ $[M]^+$ 460.1335 Found 460.1321.

1.2.2. (+)-(1*R*,2*S*,*S*s)-*tert*-Butyl 1-dimethylphosphono-2-*p*-tolylsulfinyl-2-acetylcyclopropane carboxylate (12)

The crude product was purified by chromatography (chloroform), Oil; Yield 40 %; $[\alpha]_D^{20}$ –10.2 (*c* = 1.6 acetone) δ: ^{31}P NMR (81 MHz, $CDCl_3$) δ: 19.4 ppm; ^{1}H NMR (500 MHz, $CDCl_3$) δ: 1.40 (s, 9H, COC(C$\underline{H}_3$)$_3$), 2.13 (dd, J_{HH} = 5.6 Hz, J_{PH}= 16.2 Hz, 1H, C$\underline{H}_{cis}$), 2.22 (s, 3H) 2.39 (s, 3H, $C_6H_4C\underline{H}_3$), 2.43 (dd, J_{HH} = 5.6 Hz, J_{PH} = 10.0 Hz, 1H, C$\underline{H}_{trans}$), 3.89 and 4.07 (2xd, J_{PH} = 11.3 Hz, 6H, POC$\underline{H}_3$), 7.30 and 7.46 (A_2B_2, 4H, $C_6\underline{H}_4CH_3$) ppm; ^{13}C NMR (125 MHz, $CDCl_3$) δ: 18.1, 21.1 ($C_6H_4\underline{C}H_3$), 27.2 (COC($\underline{C}H_3$)$_3$), 31.4, 34.7 (d, J_{CP} = 176.2 Hz), 53.4 (d, J_{CP} = 6.0 Hz, PO$\underline{C}H_3$), 54.2 (d, J_{CP} = 6.0 Hz, PO$\underline{C}H_3$), 58.6 (d, J_{CP} = 4.8 Hz, $\underline{C}$SO), 83.6 ($\underline{C}$OC(CH$_3$)$_3$), 124.0, 129.8, 137.5, 141.6, 163.5 (d, J_{CP} = 5.1 Hz), 195.3 ($\underline{C}$(O)CH$_3$) MS(EI) 430; HRMS (EI) *m/z* calcd for $C_{19}H_{27}O_7PS$ $[M]^+$ 430.1215 Found 430.1216.

1.3. Reaction of $PhSiH_3$ with esters

1.3.1. (1*R*,1*R*,*S*S)-Diethyl 2-hydroxymethyl-1-*p*-tolylsulfinylcyclopropylphosphonate (5a)

(*Procedure 1*) To diethyl 1-*p*-tolylsulfinyl-2-carboethoxycyclopropane phosphonate (**1,** (0.17 g 0.5 mmol) 2 mL of phenylsilane and 1.12 mg (4 mol %) of solid KOH was added and the mixture was stirred vigorously at room temperature overnight. Excess of phenysilane was removed by vacuum. A mixture of 3 mL THF, 1 mL of MeOH and a few drops of 10% HCl were added to decompose polymeric phosphorus intermediate product. Solid K_2CO_3 was then added until pH 7 and the solvent was evaporated. To the residue 3 mL of hexane were added and the mixture was stirred for 2 hours, until a white solid precipitated. Hexane solution was decanted and evaporated in vacuum affording crude **5a**, purified by column chromatography (ethyl acetate).

White crystal; yield 60 % (70 mg); m.p. 114–116 ^{0}C; $[\alpha]_D^{20}$ + 61.0 (0.21 acetone); ^{31}P NMR (81 MHz, $CDCl_3$) δ: 22.6 ppm; ^{1}H NMR (500 Mz, $CDCl_3$) δ: 1.03 (ddd, J_{HH} = 5.6, 7.1 Hz, J_{PH}= 11.5 Hz, 1H), 1.36 (t, J_{HH} = 7.1 Hz, 3H, POCH$_2$C$\underline{H}_3$), 1.40 (t, J_{HH} = 7.1 Hz, 3H, POCH$_2$C$\underline{H}_3$), 1.62 (ddd, J_{HH} = 5.6, 9.1 Hz, J_{PH}= 15.5 Hz, 1H, C$\underline{H}$), 2.43 (s, 3H, $C_6H_4C\underline{H}_3$),

White crystal; yield 60 % (70 mg); m.p. 114-116 ^{0}C; $[\alpha]_D^{20}$ + 61.0 (0.21 acetone); ^{31}P NMR (81 MHz, $CDCl_3$) δ: 22.6 ppm; ^{1}H NMR (500 Mz, $CDCl_3$) δ: 1.03 (ddd, J_{HH} = 5.6, 7.1 Hz, J_{PH}= 11.5 Hz, 1H), 1.36 (t, J_{HH} = 7.1 Hz, 3H, $POCH_2C\underline{H}_3$), 1.40 (t, J_{HH} = 7.1 Hz, 3H, $POCH_2C\underline{H}_3$), 1.62 (ddd, 1H, J_{HH} = 5.6, 9.1 Hz, J_{PH}= 15.5 Hz, $C\underline{H}$), 2.43 (s, 3H, $C_6H_4C\underline{H}_3$), 2.47-2.53 (m, 1H), 3.44-3.50 (m, 1H, OH), 3.85-3.90 (m, 1H), 3.98-4.03 (m, 1H), 4.17-4.30 (m, 4H, $POC\underline{H}_2CH_3$), 7.35 and 7.45 (A_2B_2, 4H, $C_6\underline{H}_4CH_3$) ppm; ^{13}C NMR (125 MHz, $CDCl_3$) δ: 16.4 (d, J_{PC} = 6.3 Hz, $POCH_2\underline{C}H_3$), 17.8, 21.5 ($\underline{C}H_3C_6H_4$), 33.8, 43.1 (d, J_{CP} = 179.8 Hz), 60.4 ($\underline{C}H_2OH$), 63.1 (d, J_{CP} = 5.1 Hz, $PO\underline{C}H_2CH_3$), 63.8 (d, J_{CP} = 6.3 Hz, $PO\underline{C}H_2CH_3$), 125.0, 130.0, 138.3. 141.8 ppm.; MS(EI) 346; HRMS (EI) *m/z* calcd for $C_{15}H_{23}O_5SP$ $[M]^+$ 346.1004 Found 346. 1010.

1.3.2. (1*R*, 1*S*, S_S) Diethyl-1-hydroxymethyl-2-*p*-tolylsulfinylcyclopropylphosphonate (6a)

(*Procedure 1*)

Yellowish oil; yield 67%; $[\alpha]_D^{20}$ +28.5 (0.2 acetone); ^{31}P NMR (81 MHz, $CDCl_3$) δ: 26.1 ppm; ^{1}H NMR (500 MHz, $CDCl_3$) δ: 1.31 and 1.33 (2xt, J_{HH} = 7.1 Hz, 6H, $POCH_2C\underline{H}_3$), 1.53 (ddd, J_{HH} = 5.7, 8.6 Hz, J_{PH} = 16.5 Hz, 1H, $C\underline{H}_{cis}$), 1.83 (ddd, J_{HH} = 5.9, 11.9 Hz, J_{PH} = 12.7, 1H, $C\underline{H}_{trans}$), 2.47 (s, 3H, $C_6H_4C\underline{H}_3$); 2.78 (ddd, J_{HH} = 5.9, 8.6 Hz, J_{PH} = 15.1 Hz, 1H, $C\underline{H}S$); 3.03-3.28 (m, 1H, OH), 3.89-3.97 (m, 1H, $CH\underline{H}OH$), 4.0-4.14 (m, 4H, $POC\underline{H}_2CH_3$), 4.30 (dd, J_{HH} = 11.9, 11.9 Hz, 1H, $C\underline{H}HOH$), 7.37 and 7.69 (A_2B_2, 4H, $C_6\underline{H}_4CH_3$)ppm; ^{13}C NMR (125 MHz, $CDCl_3$) δ: 14.1, 16.1 (d, J_{CP} = 6.3 Hz, $POCH_2\underline{C}H_3$), 21.4 ($C_6H_4\underline{C}H_3$), 26.2 (d, J_{CP} = 181.0 Hz), 43.4, 60.2, 62.6 (d, J_{CP} = 6.3 Hz, $PO\underline{C}H_2$), 62.8 (d, J_{CP} = 6.1 Hz, $PO\underline{C}H_2$), 124.5, 125.6, 129.3, 130.0, 140.8, 142.1 ppm; MS(EI) 346; HRMS (EI) *m/z* calcd for $C_{15}H_{23}O_5PS$ $[M]^+$ 346.1004 Found 346.1008.

1.3.3. (+)-(1*S*,2*R*)-*tert*-Butyl 1-dimethylphosphono-2-carboethoxycyclopropane carbo-xylate (7a)

(*Procedure 2*) To a stirred solution of (+)-(1*R*,2*S*,S_s)-*tert*-Butyl 1-dimethylphosphono-2-*p*-tolylsulfinyl-2-carboethoxycyclopropane carboxylate (**4**, 0.16 g, 0.35 mmol) in anhydrous THF (5 mL) phenylsilane (0.22 mL, 1.8 mmol) and solid KOH (0.016 g, 0.28 mmol) were added. The mixture was stirred at room temperature for 2 hours. Then, the solvent was evaporated and the residue was dissolved in 20 mL of hexane and stirred for 1 hour. The

decanted solution was evaporated and the crude product was purified by chromatography (hexane/acetone 4:1).

Yellowish oil; yield 88%; $[\alpha]_D^{20}$ + 16.6 (2.4 acetone); ^{31}P NMR (81 MHz, $CDCl_3$) δ: 23.7 ppm; ^{1}H NMR (500 Mz, $CDCl_3$) δ: 1.28 (t, J_{HH} = 7.2 Hz, 3H, $CO_2CH_2C\underline{H}_3$), 1.45 (s, 9H, $COC(C\underline{H}_3)_3$), 1.65 (ddd, J_{HH} = 4.5, 8.5 Hz, J_{PH}= 16.5 Hz, 1H, $C\underline{H}_{cis}$), 1.88 (ddd, J_{HH} = 4.5, 6.5 Hz, J_{PH} = 13.5 Hz, 1H, $C\underline{H}_{trans}$), 2.44 (ddd, J_{HH} = 6.5, 8.5 Hz, J_{PH}= 16.0 Hz, 1H, $C\underline{H}_{cis}$), 3.82 (d, J_{PH} = 11.0 Hz, 3H, $POC\underline{H}_3$), 3.85 (d, J_{PH} = 11.0 Hz, 3H, $POC\underline{H}_3$), 4.12-4.23 (m, 2H, $CO_2C\underline{H}_2CH_3$) ppm; ^{13}C NMR (125 MHz, $CDCl_3$) δ: 14.2 ($CO_2CH_2\underline{C}H_3$) 15.8 ($\underline{C}H_2C$), 24.5, 27.8 ($COC(\underline{C}H_3)_3$), 28.5 (d, J_{CP} = 176.1 Hz), 53.6 (d, J_{CP} = 5.1 Hz, PO$\underline{C}$H3), 53.7 (PO$\underline{C}$H3), 61.5 ($CO_2\underline{C}H_2CH_3$), 82.5 ($\underline{C}OC(CH_3)_3$), 164.6 (d, J_{CP} = 4.5 Hz), 169.2 (d, J_{CP} = 3.9 Hz); MS(CI) 323; HRMS (CI) *m/z* calcd for $C_{13}H_{24}O_7P$ $[M+1]^+$ 323.1250 Found 323.1260.

1.3.4. Methyl 2-phenylpropanoate (11)

Reaction performed according to *Procedure 2*.

Colourless oil yield 75%.^{1}H NMR (500 MHz, $CDCl_3$) δ: 1.48 (d, J = 7.2 Hz, 3H, $C\underline{H}_3$), 3.63 (s, 3H, $OC\underline{H}_3$), 3.70 (q, J_{HH}= 7.2 Hz, 1H, C$\underline{H}$), 7.20-7.31 (m, 5H, $C_6\underline{H}_5$) ppm; ^{13}C NMR (125 MHz, $CDCl_3$) δ: 18.6; 45.4, 52.1, 127.2, 127.5, 128.6, 140.6, 175.2 ppm; MS(EI) 164; HRMS(EI) *m/z* calcd for $C_{10}H_{12}O_2$ $[M]^+$ 164.0835 Found 164.0837 (in accordance with ref.10).

1.4. Reaction of $PhSiH_3$ with ketones

1.4.1. (+)-(1*S*,2*R*)-*tert*-Butyl 1-dimethylphosphono-2-acetylcyclopropane carboxylate (13)

To a stirred solution of (+)-(1*R*,2*S*,*S*s)-*tert*-butyl 1-dimethylphosphono-2-*p*-tolylsulfinyl-2-acetylcyclopropane carboxylate (**12**, 0.086 g, 0.2 mmol) in anhydrous THF (2 mL) phenylsilane (1 mmol, 0.12 mL) and (1.1 mg, 10 mol %) of solid KOH were added The reaction was stirred at room temperature for 5 hours. Excess of phenylsilane was removed by vacuum and 3 mL of hexane was added to the residue and the mixture was stirred next 2 hours, when white solid precipitated. Hexane solution was decanted and evaporated by vacuum affording crude mixture of **13** and **14**. Crude product (0.07 mmol) was dissolved in CH_2Cl_2 and the CrO_3 in pyridyne (0.7 mmol) was added. The mixture was stirred at room temperature and the reaction progress was checked by ^{31}P NMR spectrum. When the reaction

was finished CH_2Cl_2 was evaporated. The crude brown solid was dissolved in diethyl ether and filltred through Celite. The filtrate was concentrated and the crude product was purified by chromatography ($CHCl_3$).

Yellowish oil; yield 46% $[\alpha]_D^{20}$ +28.7 (6.9 acetone); ^{31}P NMR (81 MHz, $CDCl_3$) δ: 23.9 ppm; ^{1}H NMR (500 Mz, $CDCl_3$) δ: 1.44 (s, 9H, COC(C$\underline{H}_3$)$_3$), 1.61 (ddd, J_{HH} = 4.5, 8.0 Hz, J_{PH}= 16.5 Hz, 1H, C$\underline{H}_{cis}$), 1.91 (ddd, J_{HH} = 4.5, 6.5 Hz, J_{PH} = 14.0 Hz, 1H, C$\underline{H}_{trans}$), 2.35 (s, 3H, C(O)C$\underline{H}_3$), 2.67 (ddd, J_{HH} = 6.5, 8.5 Hz, J_{PH}= 16.5 Hz, 1H, C$\underline{H}_{cis}$), 3.82 (d, J_{PH} = 11.0 Hz, 3H, POC$\underline{H}_3$), 3.85 (d, J_{PH} = 11.0 Hz, 3H, POC$\underline{H}_3$) ppm; ^{13}C NMR (125 MHz, $CDCl_3$) δ: 16.6 (d, J_{CP} = 3.3 Hz), 27.7 (COC($\underline{C}H_3$)$_3$), 30.7 (d, J_{CP} = 2.5 Hz), 31.5 (d, J_{CP} = 176.2 Hz), 31.9, 53.6 (d, J_{CP} = 6.5 Hz, PO$\underline{C}H_3$), 53.7 (d, J_{CP} = 6.5 Hz PO$\underline{C}H_3$), 82.6 ($\underline{C}$OC(CH_3)$_3$), 164.7 (d, J_{CP} = 5.2 Hz, CO_2), 202.4 (d, J_{CP} = 3.0 Hz, C=O) ppm; MS(ESI) 315; HRMS (ESI) *m/z* calcd. for $C_{12}H_{21}O_6NaP$ $[M+Na]^+$ 315.0973 Found 315.0966.

1.4.2. Methyl ethyl ketone (17)

To the solution of methyl 2-(*p*-tolylsulfinyl)ethyl ketone **15** (106 mg, 0.5 mmol) in 5 mL of ethyl ether, phenylsilane (0.22 mL, 1.8 mmol) and solid KOH (1.12 mg, 4 mol %) were added. The reaction mixture was stirred vigorously for 12 hours. The crude mixture was distilled carefully affording the ketone and the remaining solvent. Due to the relative low boiling point of the obtained ketone, it was transformed to a hydrazone by addition of phenylhydrazine hydrochloride (80 mg, 0.5 mmol) and sodium acetate (45 mg, 0.5 mmol) in ethanol solution 10 mL. The mixture was stirred vigorously overnight. Solvent was evaporated and the crude product was purified by column chromatography using diethyl ether as eluent.

1.4.3. 2-Butanone 2-phenylhydrazone

^{1}H NMR (200 MHz, $CDCl_3$) δ: 1.14 (t, J_{HH}= 7.4 Hz, 3H, C$\underline{H}_3$), 1.84 (s, 3H, C$\underline{H}_3$), 2.32 (q, J_{HH}= 7.4 Hz, 2H, C$\underline{H}_2$), 6.81, 7.02 and 7.23 (m, 5H, $C_6\underline{H}_5$), 7.40 (s, 1H, N$\underline{H}$) ppm.

The phenylhydrazone obtained of commercial methyl ethyl ketone gave the same spectrum.

1.4.4. 3-(4-Methylphenyl)thio 2-butanol (18)

Reaction performed according to *Procedure 2.* The crude product obtained as mixture of diastereomers in ratio 3:1. Colourless oil; yield 97%

Major diastereomer ^{1}H NMR (500 MHz, $CDCl_3$) δ: 1.20 (d, J_{HH} = 6.2 Hz, 3H, $C\underline{H}_3$), 1.23 (d, J_{HH} = 7.1Hz, 3H, $C\underline{H}_3$), 2.31 (s, 3H, $C_6H_4C\underline{H}_3$), 2.76-79 (m, 1H, $CH\underline{H}OH$), 2.94 (dq, J_{HH} = 7.1, 7.1 Hz, 1H, $C\underline{H}$); 3.57 (dd, J_{HH} = 6.2, 6.2 Hz, 1H, $C\underline{H}$), 7.09 and 7.32 (A_2B_2, 4H, $C_6\underline{H}_4CH_3$) ppm; ^{13}C NMR (125 MHz, $CDCl_3$) δ: 17.6 ($\underline{C}H_3$), 19.4 ($\underline{C}H_3$), 21.4 ($C_6H_4\underline{C}H_3$), 52.8 ($\underline{C}H_2$), 69,7 ($\underline{C}HOH$), 129.7, 129.8, 133.9, 137.9 ppm.

Minor diastereomer ^{1}H NMR (500 MHz, $CDCl_3$) δ: 1.15 (d, J_{HH} = 6.4 Hz, 3H, $C\underline{H}_3$), 1.20 (d, J_{HH} = 7.1 Hz, 3H $C\underline{H}_3$), 2.31 (s, 3H, $C_6H_4C\underline{H}_3$), 2.76-79 (m, 1H, $CH\underline{H}OH$) 3.19 (dq, J_{HH} = 3.1, 7.1 Hz, 1H, $C\underline{H}$), 3.69-3.84 (m, 1H, $C\underline{H}$), 7.09 and 7.32 (A_2B_2, 4H, $C_6\underline{H}_4CH_3$) ppm; ^{13}C NMR (125 MHz, $CDCl_3$) δ: 14.2 ($\underline{C}H_3$), 19.2 ($\underline{C}H_3$), 21.2 ($C_6H_4\underline{C}H_3$), 51.9 ($\underline{C}H_2$), 67,7 ($\underline{C}HOH$), 129.0, 130.4, 133.0, 137.6 ppm; Anal. Calcd for $C_{11}H_{16}OS$: C 67.3; H 8.22; S 16.33. Found: C 67.46; H 8,24; S 16.19.

1.4.5. 1-(4-Bromophenyl)-2-(*p*-tolylsulfinyl)ethan-1-ol (21)

Reaction performed according to *Procedure 2:* Crude **21** formed as mixture of diastereomers in 1.2:1 ratio separated by chromatography (petroleum ether / isopropyl alcohol 40:1).
Major diastereomer **21a**: White crystals; yield 49%; m. p.127-129 ^{o}C; ^{1}H NMR (500 MHz, $CDCl_3$) δ: 2.41 (s, 3H, $C_6H_4C\underline{H}_3$); 2.88 (dd, J_{HH} = 2.5, 13.3 Hz, 1H, $C\underline{H}H$), 3.12 (dd, J_{HH} = 10.0, 13.3 Hz, 1H, $CH\underline{H}$), 4.47 (m, 1H, $O\underline{H}$), 5.35 (d, J_{HH} = 9.4 Hz, 1H, $C\underline{H}OH$), 7.26, 7.30, 7.46 and 7.54 (A_2B_2, 8H, $C_6\underline{H}_4CH_3$ and $C_6\underline{H}_4Br$) ppm; ^{13}C NMR (125 MHz, $CDCl_3$) δ: 21.4 ($C_6H_4\underline{C}H_3$), 62.6 ($\underline{C}H_2$), 70.7 ($\underline{C}HOH$), 121.9, 123.8, 127.4, 130.2, 131.7, 140.1, 142.3 ppm; Anal. Calcd for $C_{15}H_{15}BrO_2S$: C 53.11; H 4.46; S 9.45. Found: C 53.33; H 4.54; S 9.32.
Minor diastereomer **21b.** White crystals; yield 48%; m.p.148-151°C. ^{1}H NMR (500 MHz, $CDCl_3$) δ: 2.43 (s, 3H, $C_6H_4C\underline{H}_3$), 2.78 (dd, J_{HH} = 1.0, 13.5 Hz, 1H, $C\underline{H}H$), 3.21 (dd, J_{HH} = 10.2, 13.5 Hz, 1H, $CH\underline{H}$), 4.60 (m, 1H, OH), 5.22 (d, J_{HH} = 10.2 Hz, 1H, $C\underline{H}OH$), 7.17, 7.36, 7.43 and 7.54 (A_2B_2, 8H, $C_6\underline{H}_4CH_3$ and $C_6\underline{H}_4Br$) ppm; ^{13}C NMR (125 MHz, $CDCl_3$) δ: 21.4 ($C_6H_4\underline{C}H_3$), 62.6 ($\underline{C}H_2$), 68.4 ($\underline{C}HOH$), 121.7, 124.0, 127.4, 130.3, 131.7, 141.2, 141.9 ppm; MS(ESI) 360; HRMS (ESI) *m/z* calcd for $C_{15}H_{15}BrO_2SNa$ $[M+Na]^+$ 360.9660 Found 360.9674.

1.4.6. 1-(4-Bromophenyl)-2-(*p*-tolylthio)ethan-1-ol (22)

Reaction performed according to *Procedure 2:* Crude product **22**, purified by chromatography (petroleum ether / diethyl ether 10:1). Colourless oil; yield 98%; ^{1}H NMR (500 MHz, $CDCl_3$) δ: 2.41 (s, 3H, $C_6H_4C\underline{H}_3$); 3.03 (dd, J_{HH} = 9.4, 13.9 Hz, 1H, $C\underline{H}H$), 3.09 (m, 1H, $O\underline{H}$), 3.28

(dd, J_{HH} = 3.5, 13.9 Hz, 1H, $C\underline{H}H$), 4.68 (d, J_{HH} = 9.4 Hz, 1H, $C\underline{H}OH$), 7.20, 7.26, 7.39 and 7.51 (A_2B_2, 8H, $C_6\underline{H}_4CH_3$ and $C_6\underline{H}_4Br$) ppm; ^{13}C NMR (125 MHz, $CDCl_3$) δ: 21.0 ($C_6H_4\underline{C}H_3$), 44.7, 70.7 ($\underline{C}HOH$), 121.6, 127.5, 129.9, 130.4, 131.2, 137.3, 141.1 ppm; Anal. Calcd for $C_{15}H_{15}BrOS$: C 55.74; H 4.68; S 9.92 Found: C 55.49; H 4,84; S 9.90.

2. ^{1}H and $^{13}C\{1H\}$ NMR spectra of the starting materials

(+)-(1*R*,2*S*,*S*s)-*tert*-Butyl 1-dimethylphosphono-2-*p*-tolylsulfinyl-2-carboethoxycyclopropa-ne carboxylate (4).

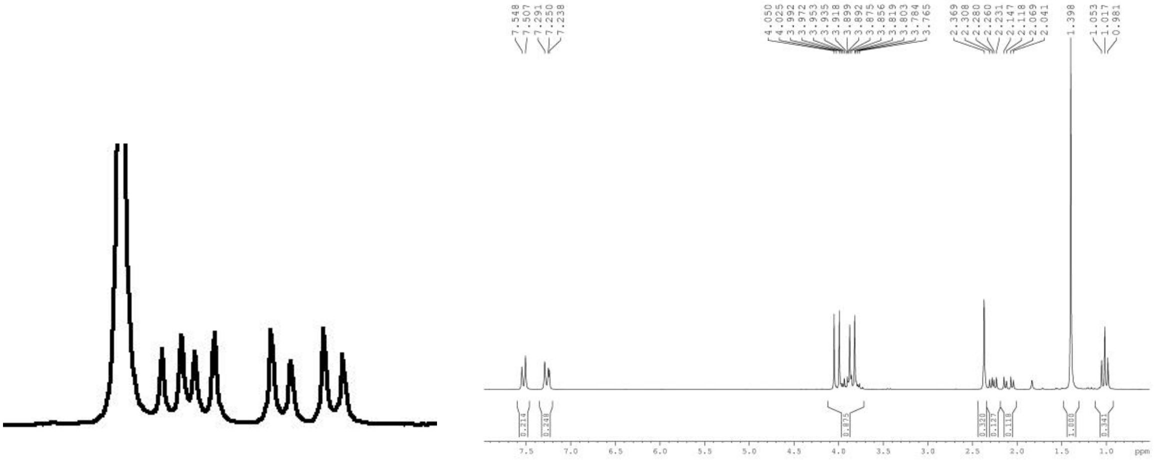

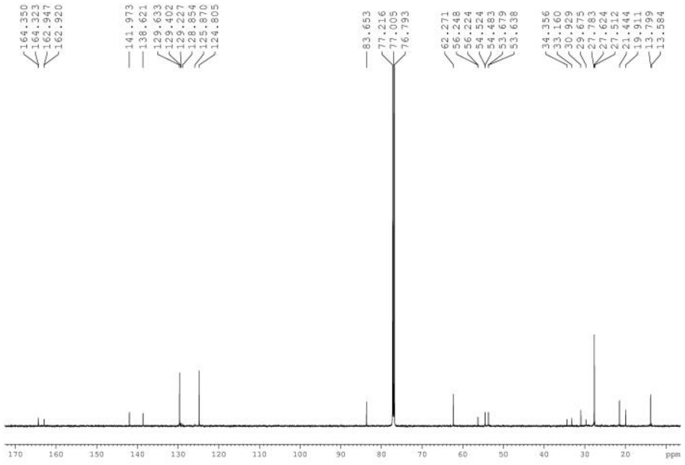

(+)-(1*R*,2*S*,*S*s)-*tert*-Butyl 1-dimethylphosphono-2-*p*-tolylsulfinyl-2-acetylcyclopropane carboxylate (12)

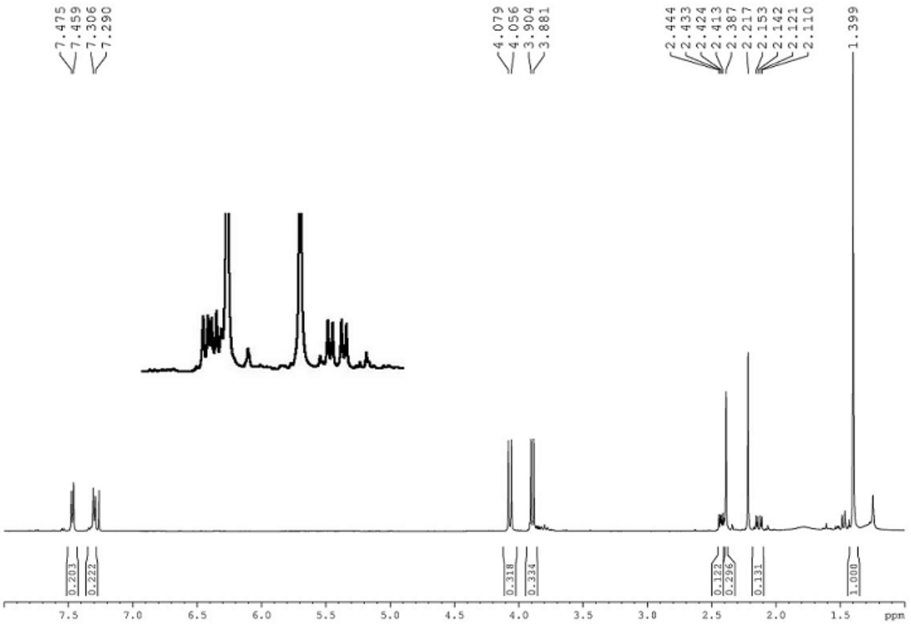

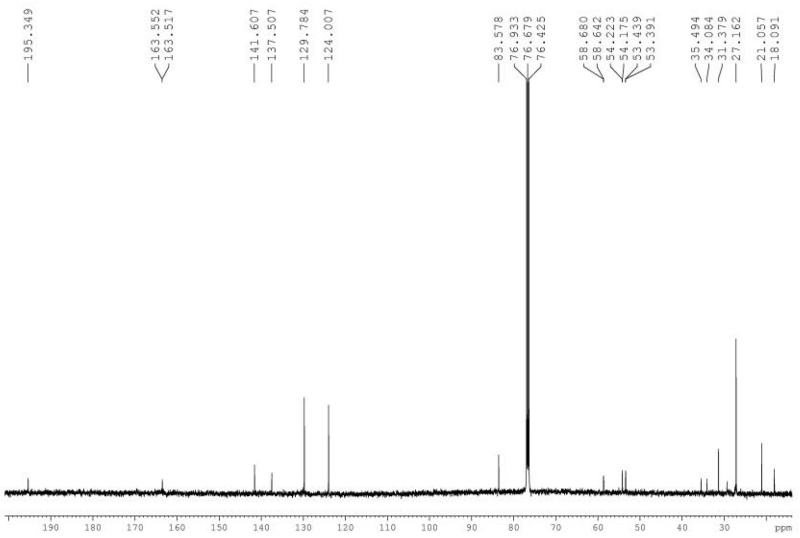

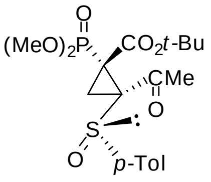

12

Methyl 2-phenyl-2-(*p*-tolylsulfinyl)acetate (9)

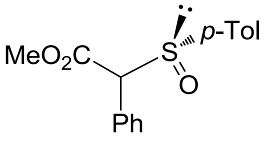

9

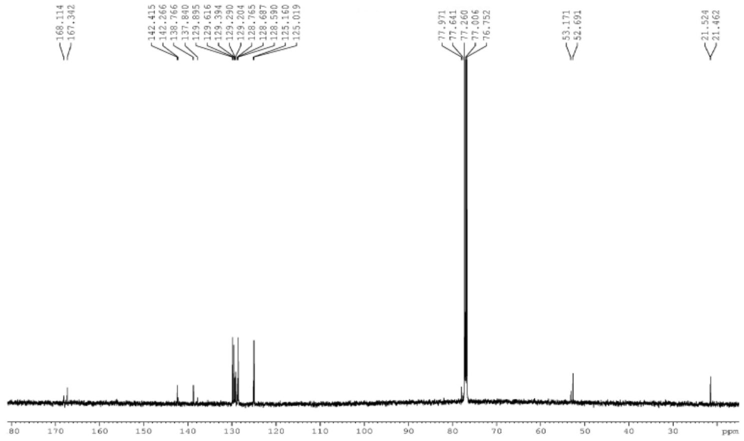

Methyl 2-phenyl-2-(*p*-tolylsulfinyl)propanoate (10)

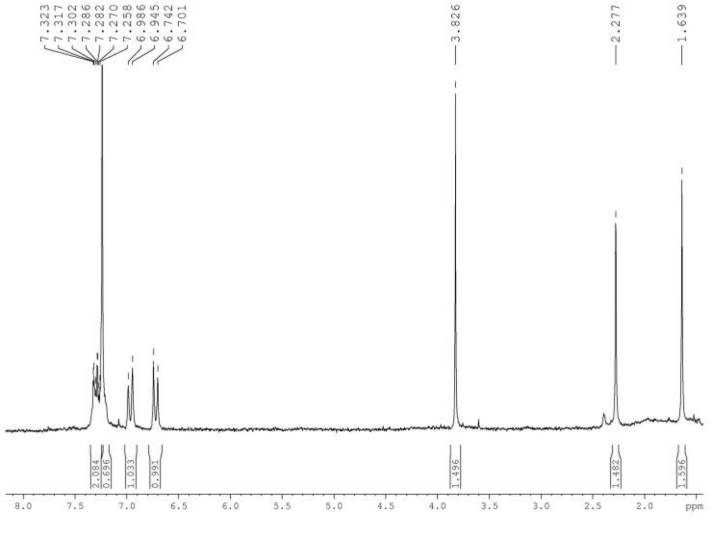

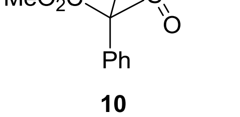

10

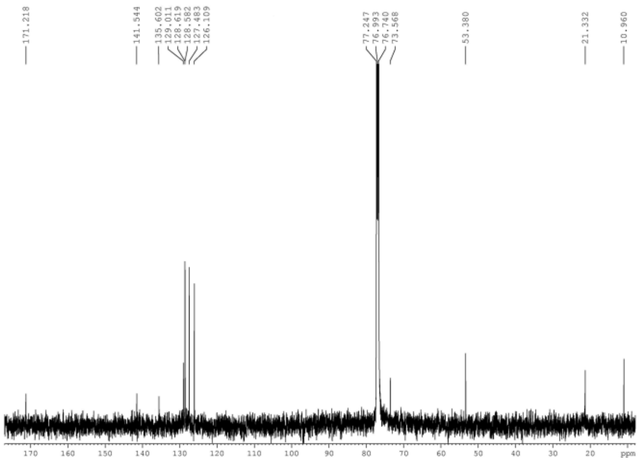
171.218
141.544
135.602
129.011
128.619
128.582
127.483
126.109
77.247
76.993
76.740
73.568
53.380
21.332
10.960
ppm

3. ^{1}H and ^{13}C{1H} NMR spectra of the products

Diethyl (1*S*,2*R*,*S*$_S$)-2-hydroxymethyl-1-*p*-tolylsulfinylcyclopropylphosphonate (5a)

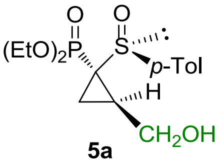

5a

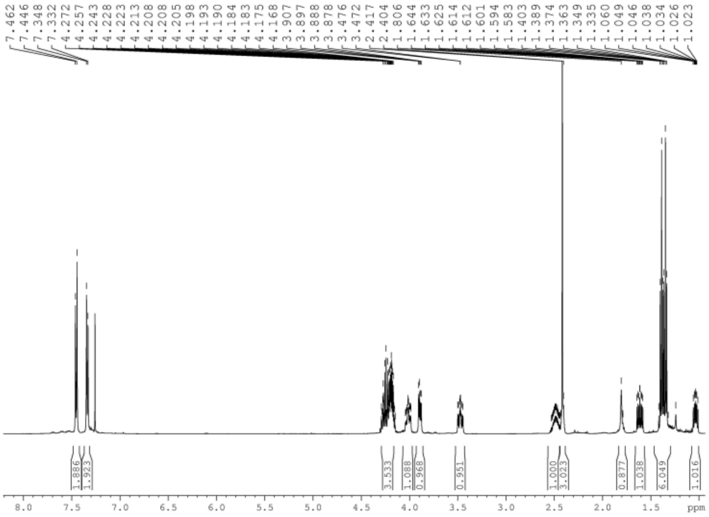

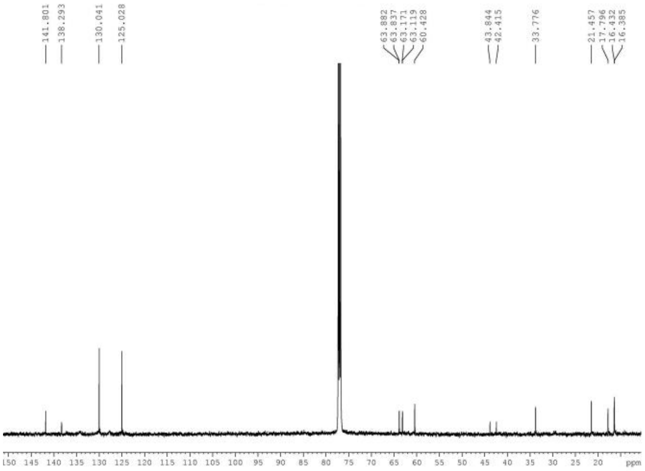

Diethyl (1*R*, 2*S*, S_S)-1-hydroxymethyl-2-*p*-tolylsulfinylcyclopropylphosphonate (6a)

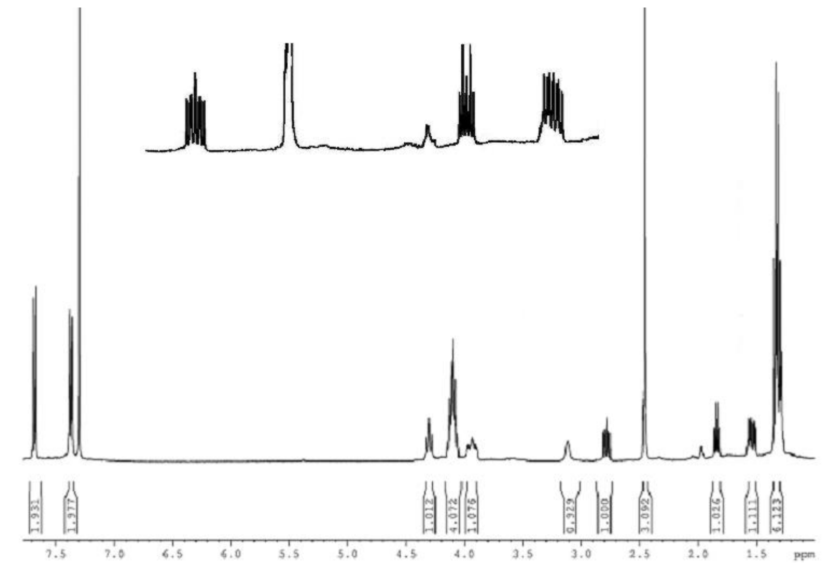

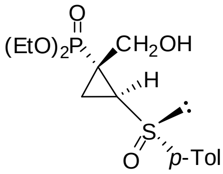

6a

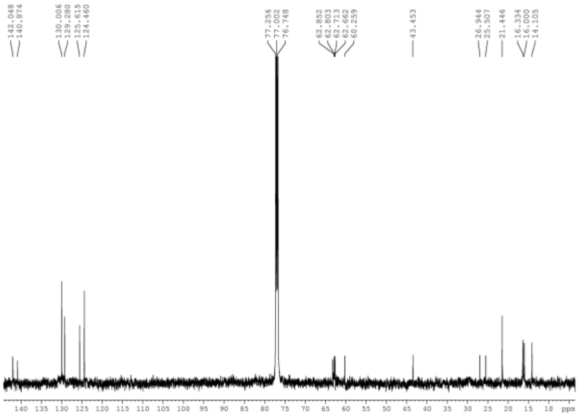

(+)-(1*S*,2*R*)-*tert*-Butyl 1-dimethylphosphono-2-carboethoxy cyclopropane carboxylate (7a).

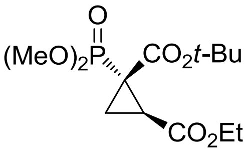

7a

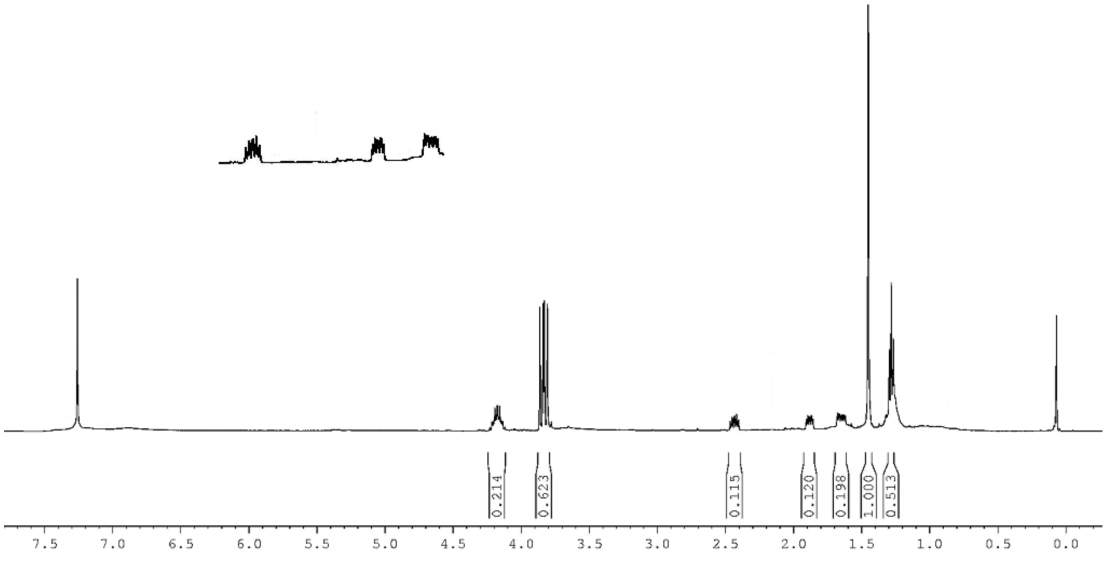

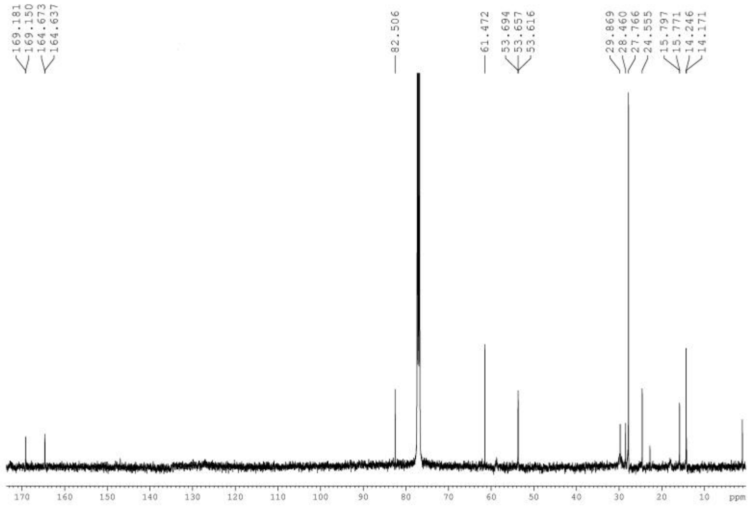

(+)-(1*S*,2*R*)-*tert*-Butyl 1-dimethylphosphono-2-acetyl cyclopropane carboxylate (13)

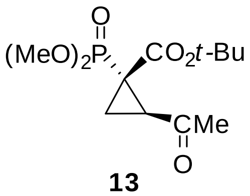

13

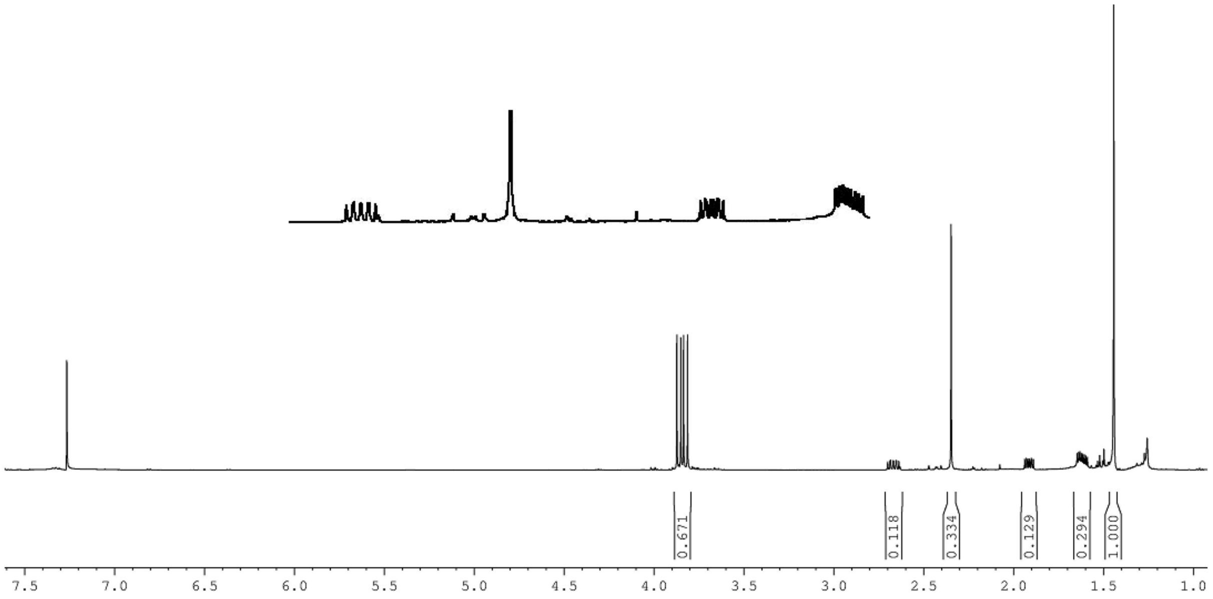

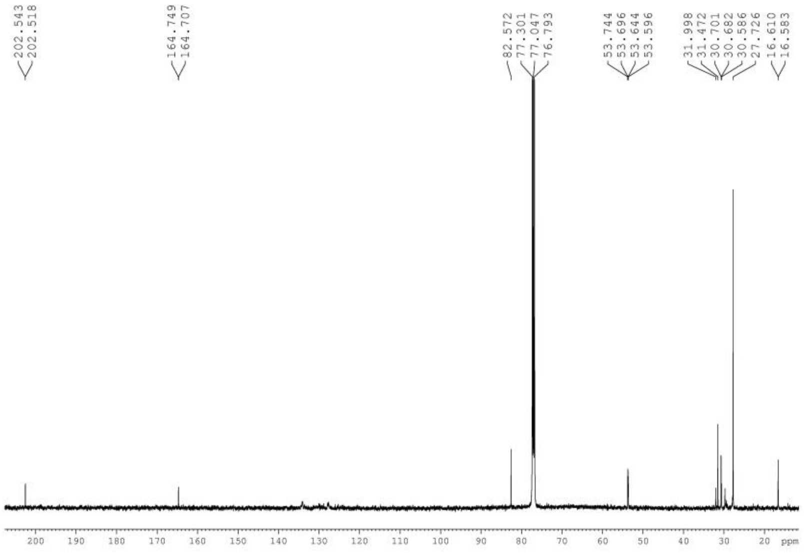

3-(*p*-Tolyl)thio 2-butanol (18)

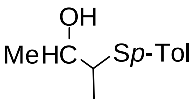

18

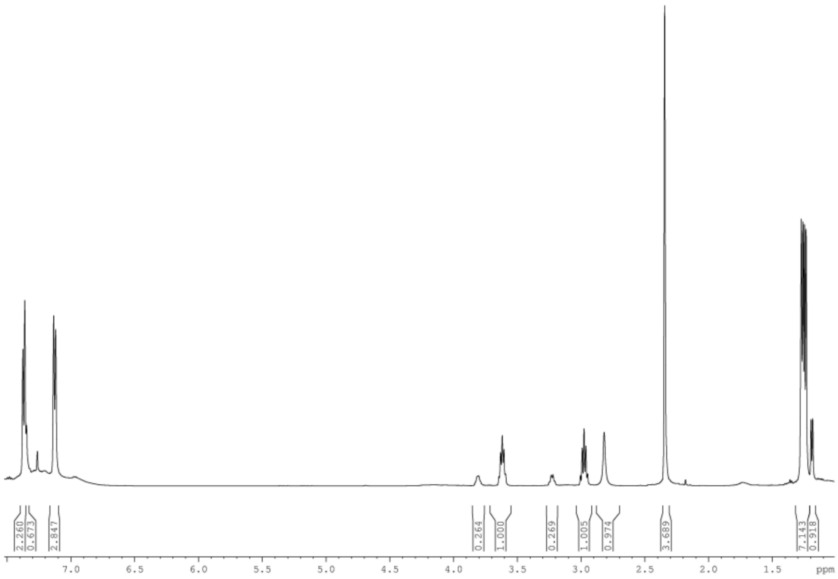

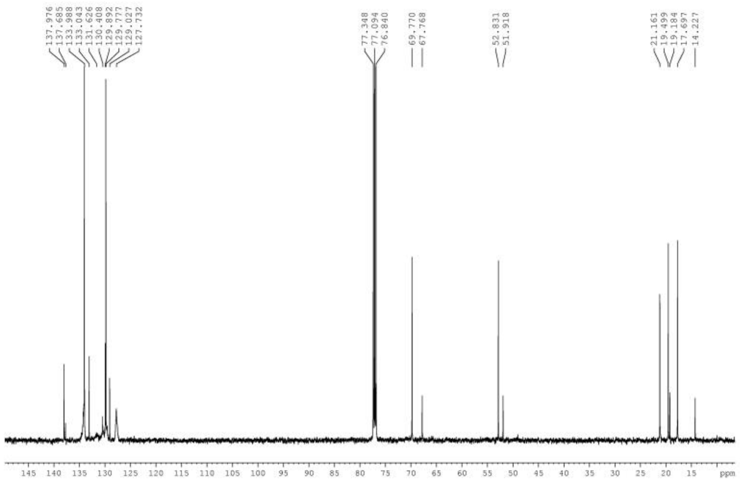

1-(4-bromophenyl)-2-(*p*-tolylsulfinyl)ethan-1-ol (21a)

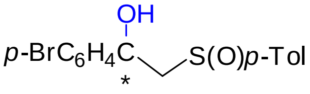

21

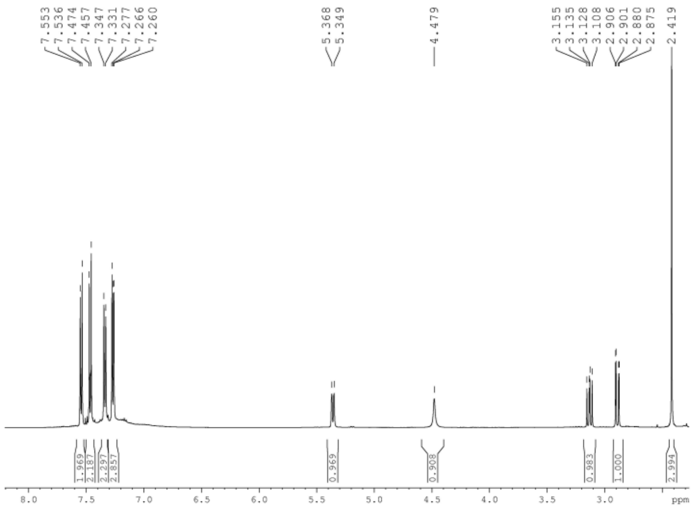

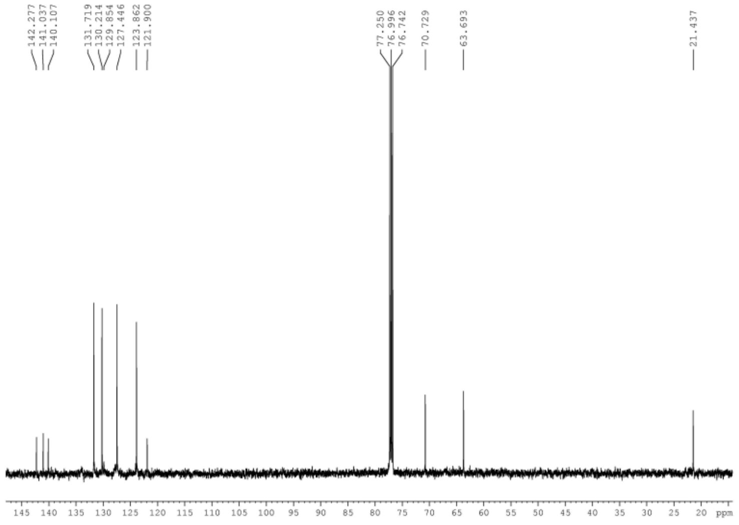

1-(4-bromophenyl)-2-(*p*-tolylsulfinyl)ethan-1-ol (21b)

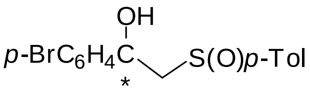

21

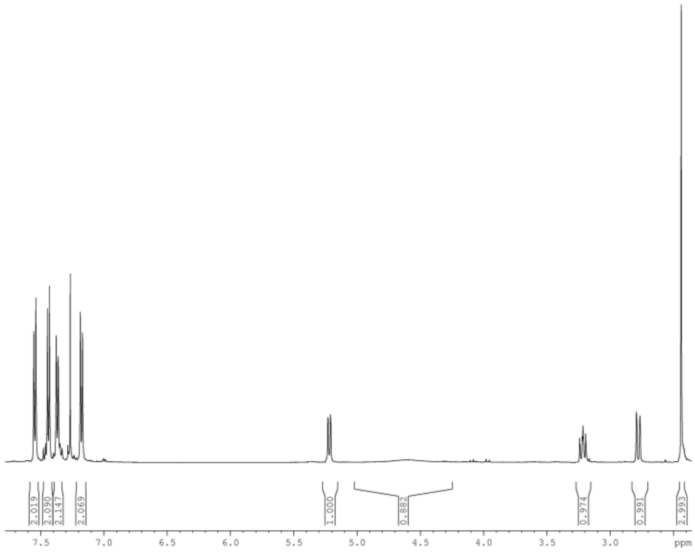

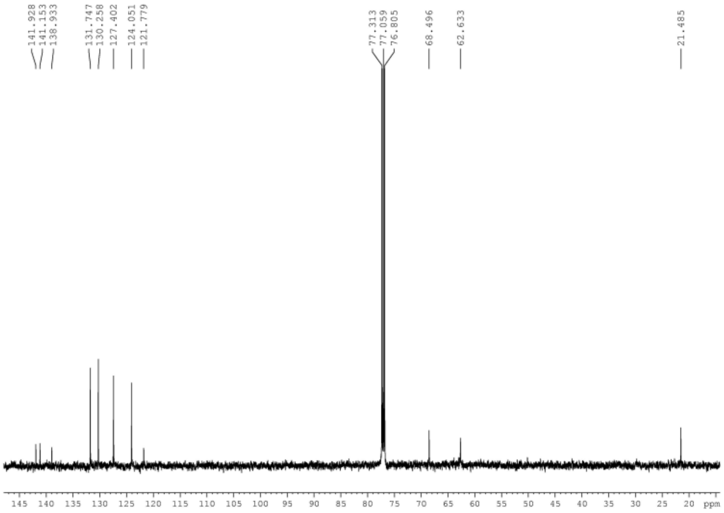

1-(4-Bromophenyl)-2-(*p*-tolylthio)ethan-1-ol (22)

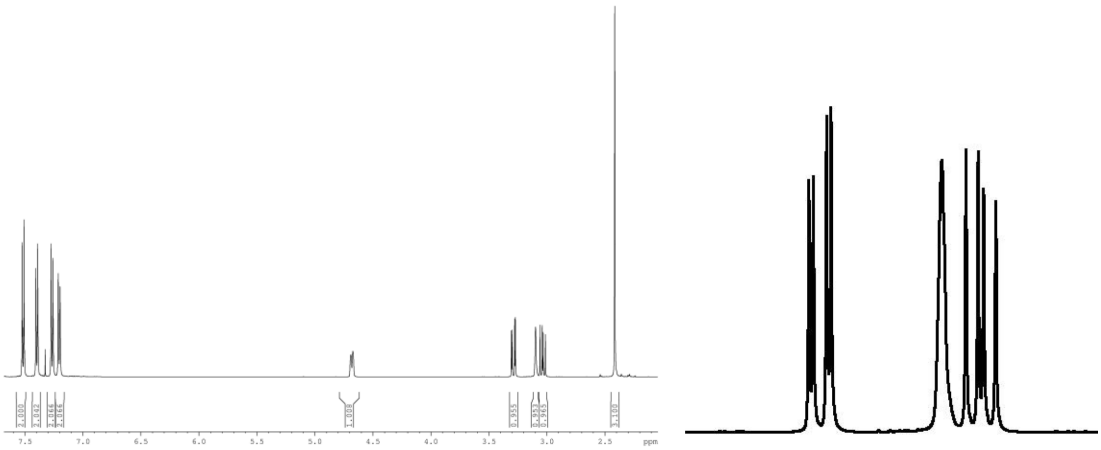

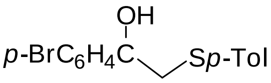

22

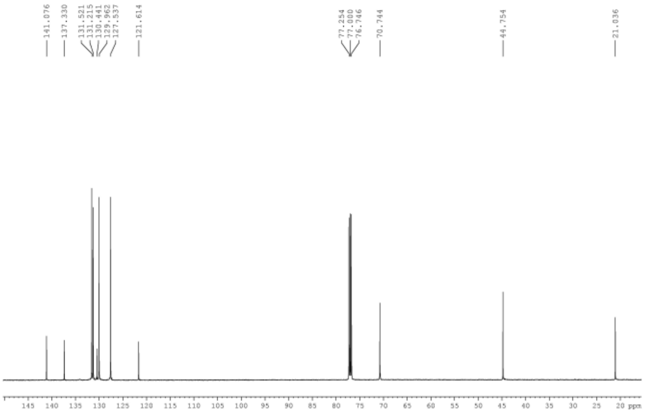

Supplement: File 1 — Experimental and analytical data and NMR spectra. [file Beilstein_J_Org_Chem-13-1513-s001.pdf]
